# Causal role of immune cells in prostate cancer: a bidirectional Mendelian-randomization analyses

**DOI:** 10.18632/aging.205942

**Published:** 2024-06-17

**Authors:** Mingyan Zhong, Xiangpeng Zhan, Fang-Ping Zhong

**Affiliations:** 1 Department of Oncology, Pingxiang Second People’s Hospital, Jiangxi, China; 2 Department of Urology, The First Affiliated Hospital, Jiangxi Medical College, Nanchang University, Jiangxi, China

**Keywords:** immunity, prostate cancer, cause association, Mendelian-randomization, GWAS

## Abstract

Background: Immune cell signatures have been implicated in cancer progression and response to treatment. However, the causal relationship between immune cell signatures and prostate cancer (PCa) is still unclear. This study aimed to investigate the potential causal associations between immune cell signatures and PCa using Mendelian randomization (MR).

Method: This study utilized genome-wide association studies (GWAS) summary statistics for PCa and immune cell signatures from publicly available datasets. MR analyses, including IVW, MR-Egger, and weighted median methods, were performed to evaluate the causal associations between immune cell signatures and PCa. Multiple sensitivity analysis methods have been adopted to test the robustness of our results.

Results: After FDR correction, our findings suggested that specific immune cell signatures, such as HLA DR on CD33+ HLA DR+ CD14dim (odds ratio [OR] = 1.47, 95% confidence interval [CI] = 1.12-1.92, p = 0.006), HLA DR on CD33+ HLA DR+ CD14− (OR = 1.32, 95% CI = 1.05-1.67, p = 0.018), and HLA DR on monocyte (OR = 1.23, 95% CI = 1.03-1.47, p = 0.021), were significantly associated with PCa. PCa had no statistically significant effect on immunophenotypes. These results remained robust in sensitivity analyses, supporting the validity of the causal associations.

Conclusions: This study provides evidence of a potential causal relationship between certain immune cell signatures and PCa. We observed that immune cell signatures involving HLA DR expression on specific cell types are associated with an increased risk of PCa.

## INTRODUCTION

Prostate cancer (PCa) was a significant global public health concern, ranking as the second most commonly diagnosed cancer and the fifth leading cause of death among men [1]. Genomics emerged as a crucial factor in the complex etiology of PCa. Notably, studies observed a higher incidence of PCa in men of African ancestry and individuals with a positive familial history, indicating genetic predisposition [2, 3]. While active surveillance was a viable option for low-risk PCa cases, locally advanced PCa often necessitated aggressive treatments such as surgery, radiotherapy, and androgen deprivation therapy (ADT). Unfortunately, once PCa metastasized, the survival rate significantly declined [2].

Recently, there has been increasing recognition of the role of immune cells in the development and progression of PCa. The tumor microenvironment is a crucial factor that influences tumor behavior and immune response. Several studies have shed light on the interaction and infiltration of immune cells within the tumor microenvironment [3–5]. These immune cells can either promote or suppress tumor growth, thus affecting the progression of PCa. One important aspect is the infiltration of immune cells into the tumor microenvironment. The presence of different immune cells, such as T cells, B cells, natural killer (NK) cells, and macrophages, within the prostate tumor has been linked to varying outcomes. For example, a high infiltration of CD8+ cytotoxic T cells has been associated with improved prognosis and increased survival in PCa patients [3]. In addition to the immune cells mentioned, other immune cells and molecules play important roles in the tumor microenvironment of prostate cancer (PCa). For example, tumor-associated macrophages (TAMs) are a type of immune cell that can have both pro-tumor and anti-tumor functions depending on their polarization. M1-polarized TAMs have anti-tumor properties and can promote immune responses against cancer cells, while M2-polarized TAMs have pro-tumor properties and can suppress immune responses and promote tumor growth. Furthermore, dendritic cells (DCs) are antigen-presenting cells that play a crucial role in initiating and regulating immune responses. In PCa, the function of DCs can be impaired, leading to a reduced ability to activate T cells and mount an effective anti-tumor immune response. Understanding the complex interactions between immune cells, tumor cells, and the microenvironment is crucial for the development of effective immunotherapeutic strategies for PCa. Targeting immunosuppressive cells and molecules, as well as enhancing anti-tumor immune responses, are potential approaches to improve the treatment outcomes for PCa patients. Additionally, combination therapies that target multiple components of the tumor microenvironment may be necessary to overcome the immunosuppressive barriers and achieve durable responses in PCa.

The Mendelian randomization (MR) design, utilizing genetic variation as an instrumental variable (IV) for exposure, has the potential to strengthen causal relationships [6]. This approach can mitigate the influence of residual confounding as genetic variation is randomly allocated during conception, independent of environmental factors and lifestyle choices. Moreover, the MR design can minimize the chance of reverse causation since genetic variation remains unaffected by the onset and progression of disease [7].

Previous observational studies have identified numerous correlations between immune cell traits and PCa, supporting the hypothesis of a relationship between them [8, 9]. In this study, a comprehensive two-sample Mendelian randomization analysis was conducted to ascertain the causal relationship between immune cell signatures and PCa.

## MATERIALS AND METHODS

### Study design

**Figure 1:**
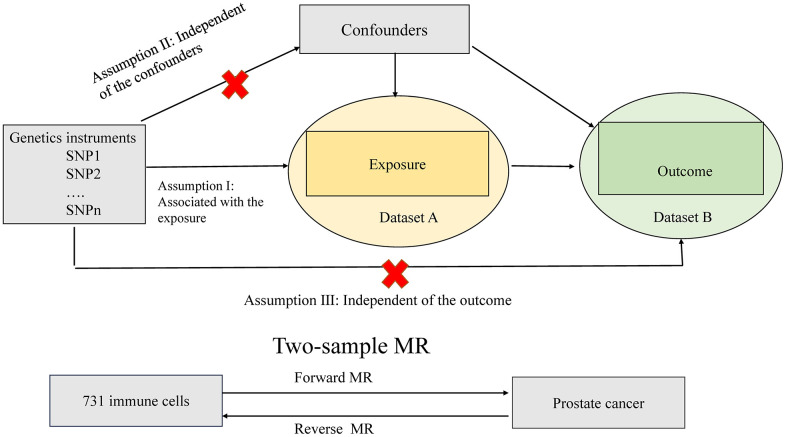
**Assumptions of the Mendelian randomization (MR) analysis for immune cell signatures and PCa.** The MR study assumes that genetic variants are associated with only immune cell signatures and not with confounders or alternative causal pathways, that is, the IVs affect the PCa only directly through immune cell signatures. IVs, instrument variables.

Figure 1 depicts the design and assumptions of this study, which utilized Mendelian randomization (MR) in a two-sample framework using genome-wide association studies (GWAS) summary statistics to explore cause association between 731 immune cell signatures and PCa. MR analysis is a gene-based approach that leverages naturally allocated genetic variants at conception to infer causal effects between exposure and outcomes. Our study relies on three key assumptions: (1) the instrumental variables, i.e., the genetic variants, should be strongly associated with the exposure; (2) the instrumental variables should not be associated with any confounding factors; and (3) there should be no direct connection between the instrumental variables and the outcome (Figure 1). Ethical approval was not required for our research as we used publicly available summary data from existing studies that had already undergone ethical review by academic ethics committees, with participants providing written informed consent. Our study adhered to the STROBE-MR guidelines, and the supporting information Supplementary Table 1 contains the corresponding checklist (Supplementary Table 1).

### Data sources and instrument selection

GWAS summary statistics for prostate cancer were acquired from the Prostate Cancer Association Group to Investigate Cancer Associated Alterations in the Genome (PRACTICAL) consortium [9]. The study conducted a GWAS on a total of 150,064 individuals of European descent, with 79,148 cases and 61,106 controls. After implementing quality control measures and imputation, a total of 20,346,368 genetic variants were analyzed.

We obtained GWAS summary statistics for various immune traits from the website https://gwas.mrcieu.ac.uk/. In total, it reported on the impact of approximately 22 million variants on 731 immune phenotypes in a population of 3,757 Sardinians [8]. These encompassed absolute cell counts (n=118), median fluorescence intensities indicating surface antigen levels (n=389), morphological parameters (n=32), and relative cell counts (n=192).

**Figure 2:**
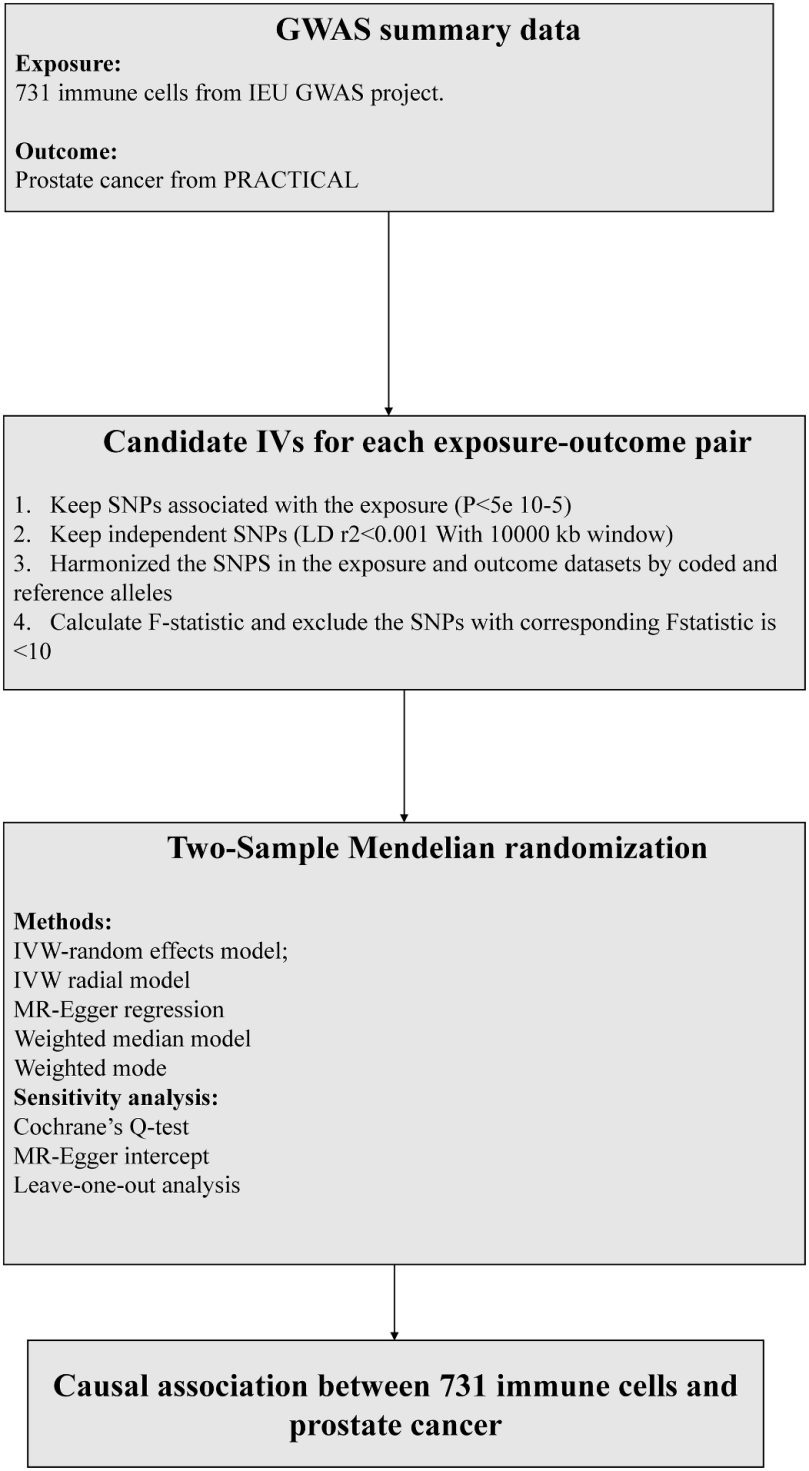
**The flow chart of the inclusion and exclusion criterion of candidate SNPs for each exposure-outcome pair.** MR, Mendelian randomization, IVW, inverse-variance weighted.

According to recent research [10], the significance level of independent variables (IVs) for each immune trait was set at 1 × 10^-5. To prune these SNPs (linkage disequilibrium [LD] r2 threshold < 0.001 within 10,000 kb distance) [11], we utilized the clumping procedure in PLINK software. As for PCa, we adjusted the significance level to 5 × 10^-8. To evaluate the strength of the instruments and avoid weak instrumental bias, we computed the proportion of phenotypic variation explained (PVE) and F statistic for each IV. IVs with low F statistics (< 10) were removed (Figure 2).

### Statistical analysis

Bidirectional univariable Mendelian randomization (MR) was conducted to investigate the causal association between 731 immune cell signatures and PCa. Instrumental single-nucleotide polymorphisms (SNPs) from the exposure data were extracted from the outcome genome-wide association studies (GWASs) to obtain the results. To ensure accurate harmonization of alleles, the SNPs were arranged in a way that the effect variants of both exposure and outcome matched the same allele [NO4]. Four MR methods were applied, namely inverse variance weighted (IVW), weighted median, MR-Egger, and Weighted mode, to evaluate the causal association between731 immune cell signatures and PCa. IVW was used to generate effect estimates as the primary outcome and could provide unbiased estimates only when there is no horizontal pleiotropy [NO5]. MR Egger regression analysis can detect and correct for directional pleiotropy but at the cost of reduced power. The P-value of the MR Egger intercept is used to indicate directional pleiotropy. Assuming that no more than 50% of MR effect estimates are due to pleiotropic SNPs, the penalized weighted median approach can provide consistent effect estimates, with weights determined by their association strength with the exposure [12]. Under the condition that more than 50% of the weight comes from valid instrumental variables (IVs) in the analysis, the weighted median model provides consistent estimates [12].

We performed several sensitivity analyses to ensure the reliability of the MR test, including the MR-Egger intercept test, Cochran’s Q test, and leave-one-out analysis. The MR-Egger intercept analysis was used to assess directional heterogeneity. A significant deviation from zero in the MR-Egger intercept indicated the presence of directional heterogeneity. Cochran’s Q statistic was employed to determine the existence of heterogeneity. If the p-value was significant, it indicated the presence of heterogeneity, and the random effects IVW MR method would be used. Leave-one-out analysis was conducted to identify any individual SNP that had a disproportionately large effect on the estimates. To account for multiple testing in our analysis, we applied False Discovery Rate (FDR) correction. Correlations with a p-value less than 0.05 were considered significant, while unadjusted p-values between 0.05 and 0.10 were regarded as suggestive. All statistical analyses were performed using the Mendelian Randomization package (version 0.4.2) and Two Sample MR package (version 0.5.5).

### Availability of data and materials

We obtained data from https://gwas.mrcieu.ac.uk/. The original contributions presented in the study are included in the article/Supplementary Material, further inquiries can be directed to the corresponding author.

## RESULTS

### Causal effect of immunophenotypes on PCa

**Figure 3:**
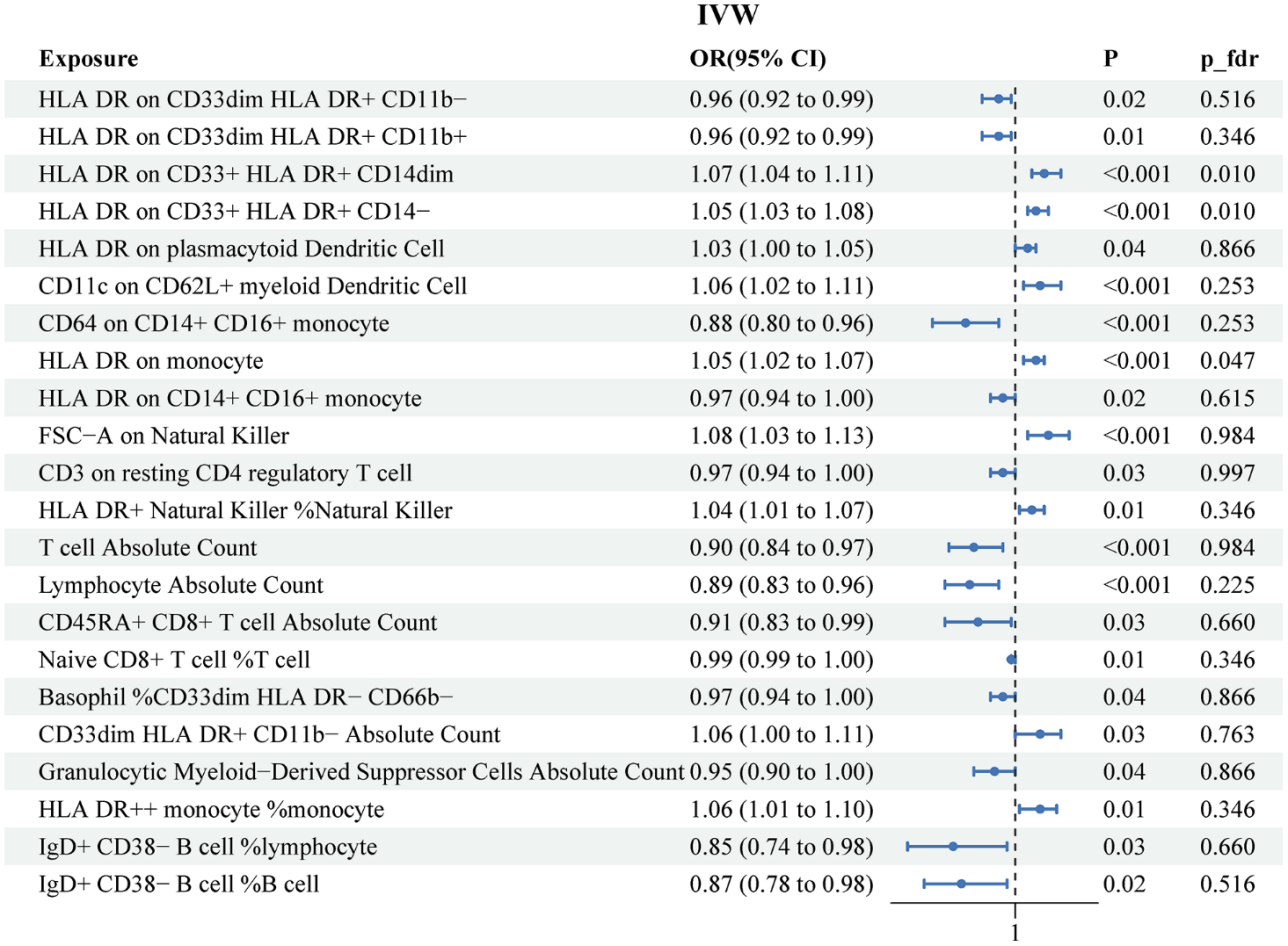
**Associations of genetically predicted immune cell signatures and the risk of prostate cancer.** SNPs, single nucleotide, polymorphisms; IVW, inverse variance weighted.

Supplementary Table 2 described detailed information on genetic instruments of 731 immune cell signatures used to explore cause effect of immunophenotypes on PCa. The MR IVW results detected 22 suggestive immunophenotypes associated with risk of PCa, which included 9 risk factors (HLA DR on CD33+ HLA DR+ CD14dim, HLA DR on CD33+ HLA DR+ CD14−, HLA DR on plasmacytoid Dendritic Cell, CD11c on CD62L+ myeloid Dendritic Cell, HLA DR on monocyte, FSC−A on Natural Killer, HLA DR+ Natural Killer %Natural Killer, CD33dim HLA DR+ CD11b− Absolute Count, HLA DR++ monocyte %monocyte) and 13 protective factors (HLA DR on CD33dim HLA DR+ CD11b−, HLA DR on CD33dim HLA DR+ CD11b+, CD64 on CD14+ CD16+ monocyte, HLA DR on CD14+ CD16+ monocyte, CD3 on resting CD4 regulatory T cell, T cell Absolute Count, Lymphocyte Absolute Count, CD45RA+ CD8+ T cell Absolute Count, Naive CD8+ T cell %T cell, Basophil %CD33dim HLA DR− CD66b−, Granulocytic Myeloid−Derived Suppressor Cells Absolute Count, IgD+ CD38− B cell %lymphocyte, IgD+ CD38− B cell %B cell) (Figure 3). The results of other three MR method were recorded in Supplementary Table 3. After FDR adjustment (PFDR<0.05), we detected risk effects of three immunophenotypes on PCa: HLA DR on CD33+ HLA DR+ CD14dim, HLA DR on CD33+ HLA DR+ CD14−, and HLA DR on monocyte. Specifically, the odds ratio (OR) of HLA DR on CD33+ HLA DR+ CD14dim on risk of PCa was 1.07 (95%CI: 1.04-1.11, P<0.001, P_fdr=0.01). The other three methods were as followed: MR Egger (OR: 1.06, 95%CI: 0.90-1.26, P=0.61), Weighted median (OR: 1.07, 95%CI: 1.03-1.11, P<0.001), Weighted mode (OR: 1.07, 95%CI: 1.03-1.11, P=0.007). The estimated OR of HLA DR on CD33+ HLA DR+ CD14− was 1.05 (95%CI: 1.03-1.08, P<0.001, P_fdr=0.01). The IVW OR of HLA DR on CD33+ HLA DR+ CD14− was 1.05 (95%CI: 1.03-1.08, P<0.001, P_fdr=0.01). Other methods results were observed as followed: MR Egger (OR: 1.04, 95%CI: 0.94-1.15, P=0.57), Weighted median (OR: 1.05, 95%CI: 1.03-1.07, P<0.001), Weighted mode (OR: 1.05, 95%CI: 1.02-1.08, P=0.007). The OR of HLA DR on monocyte was 1.05 (95%CI: 1.02-1.07, P<0.001, P_fdr=0.047). Similar results were observed from the other three methods: MR Egger (OR: 1.08, 95%CI: 1.007-1.06, P=0.016), Weighted median (OR: 1.05, 95%CI: 1.02-1.07, P<0.001), Weighted mode (OR: 1.05, 95%CI: 1.02-1.08, P=0.03).

The scatter plots for the MR significative immune cell signatures-to-PCa association were presented in Supplementary Figure 1. There was no heterogeneity for significative MR results (P for Cochrane’s Q>0.05). The analysis of the MR-Egger intercept did not detect any indication of horizontal pleiotropy. The other heterogeneity test and horizontal pleiotropy results were recorded in Supplementary Tables 4, 5). The leave-one-out sensitivity analysis suggested that the genetic prediction of estimating the significative immune cell signatures-to-PCa association was robust (Supplementary Figure 2).

### Causal effect of PCa on immunophenotypes

**Figure 4:**
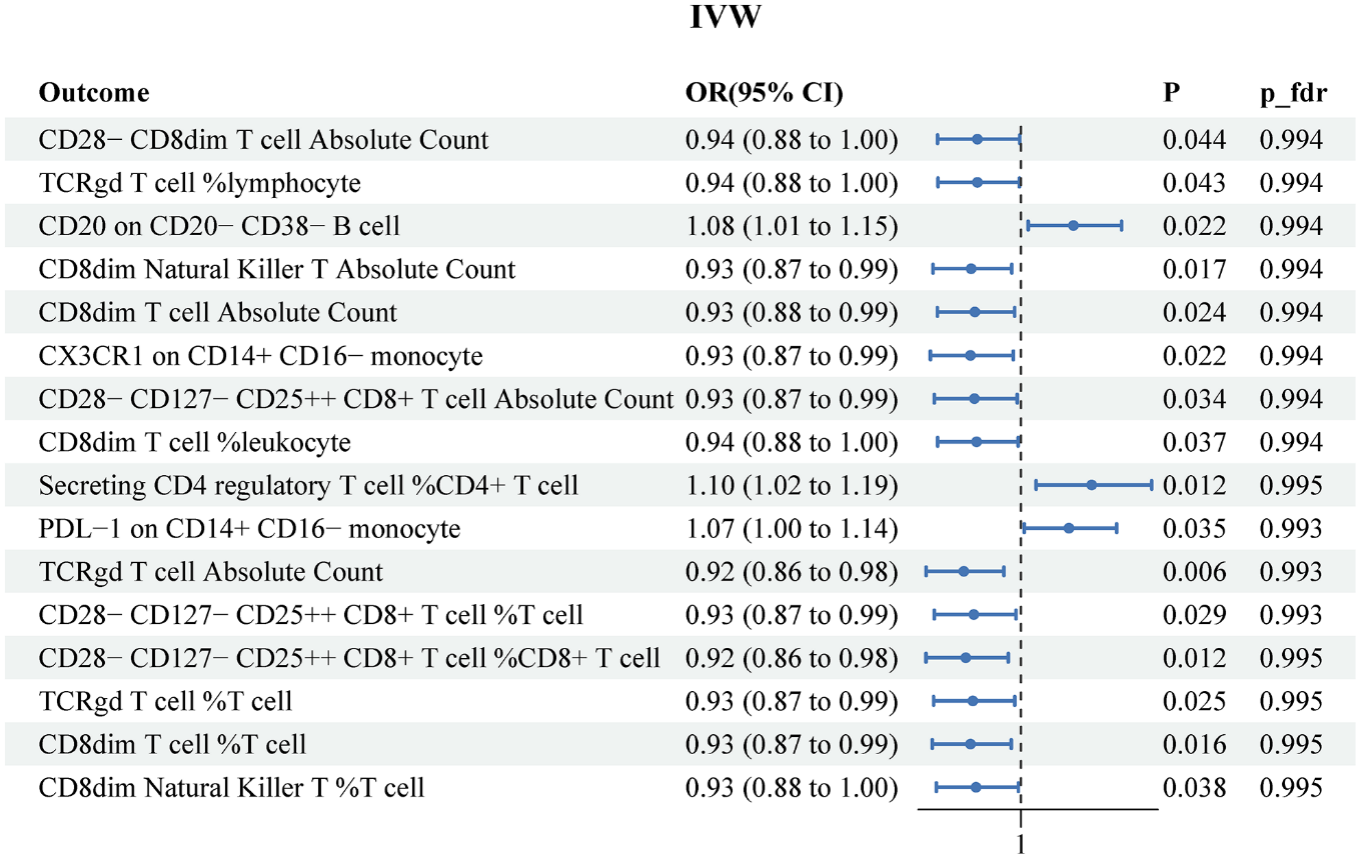
**Reverse MR results for the effects of prostate cancer on immune cell signatures.** IVW, inverse variance weighted.

The results of the MR IVW analysis indicated that PCa was correlated with 16 immune traits. These comprised of 3 risk factors (CD20 on CD20− CD38− B cell, Secreting CD4 regulatory T cell %CD4+ T cell and PDL−1 on CD14+ CD16− monocyte) and 13 protective factors (CD28− CD8dim T cell Absolute Count, TCRgd T cell %lymphocyte, CD8dim Natural Killer T Absolute Count, CD8dim T cell Absolute Count, CX3CR1 on CD14+ CD16− monocyte, CD28− CD127− CD25++ CD8+ T cell Absolute Count, CD8dim T cell %leukocyte, TCRgd T cell Absolute Count, CD28− CD127− CD25++ CD8+ T cell %T cell, CD28− CD127− CD25++ CD8+ T cell %CD8+ T cell, TCRgd T cell %T cell, CD8dim T cell %T cell, and CD8dim Natural Killer T %T cell. (Figure 4). Supplementary Table 6 recorded the results obtained from the other three MR methods. However, after conducting FDR adjustment (PFDR<0.05), none of the immune traits were found to be significant at the 0.05 level (all P_fdr>0.05). The heterogeneity test and horizontal pleiotropy test results were separately described in Supplementary Tables 7, 8.

## DISCUSSION

This study utilized MR in a two-sample framework with GWAS summary statistics to investigate the causal association between 731 immune cell signatures and PCa. The findings revealed that 22 immune cell signatures were associated with the risk of PCa, including 9 risk factors and 13 protective factors. Further analysis, after adjusting for multiple testing, identified three immune cell signatures that were significantly associated with the risk of PCa. Conversely, the study did not find a significant impact of PCa on immune cell signatures.

The advent of ICB signified the dawn of a groundbreaking era in immunooncology, where the primary focus of therapy shifted from cancer cells to immune cells [3]. Immunotherapy showed promise in the treatment of PCa, although its success was limited compared to other solid tumors [13]. PCa was considered immunologically “cold,” characterized by restricted CD8+ T cell infiltration and low tumor mutational burden (TMB). Additionally, PCa created an immunosuppressive microenvironment using supporting stromal cells, endothelial cells, and immune cells. Lifestyle factors like microbiota and diet also influenced the tumor microenvironment. Current FDA-approved immunotherapies for PCa included Sipuleucel-T, a cancer vaccine that stimulated the patient’s immune cells *ex vivo*, and pembrolizumab, an anti-PD-1 antibody that blocked immune checkpoints. However, these single-agent therapies had limited efficacy [14, 15]. To enhance the effectiveness of immunotherapy, there was a need to better understand the immune populations present within the tumor microenvironment and identify resistance mechanisms. Exploring the interaction between PCa and the extracellular matrix and stromal elements involved in chronic inflammation and immune suppression was crucial. Developing a novel immunogenomic classification strategy could guide the selection of combination immunotherapies for PCa. By combining different immunotherapeutic approaches and modulating the tumor microenvironment, it might have been possible to overcome the immunosuppressive circuits and enhance the recognition and eradication of PCa by the immune system.

In this study, we found that the risk of PCa increased with the higher proportion of HLA DR on CD33+ HLA DR+ CD14dim, HLA DR on CD33+ HLA DR+ CD14− and HLA DR on monocyte. HLA-DR, a significant molecule belonging to the major histocompatibility complex (MHC) class II family, played a vital role in the immune system. Its primary function lied in antigen presentation, wherein it facilitated the display of antigen fragments to CD4+ T cells, thereby regulating and stimulating immune responses. In the realm of prostate cancer research, a specific subtype of HLA-DR known as HLA-DR2b emerged as a critical player. Previous studies had employed mouse models that expressed HLA-DR2b to investigate prostate cancer, and intriguingly, an association between HLA-DR2b and immune responses directed against prostate-specific antigen (PSA) had been observed [11, 16]. The presence of HLA-DR2b in prostate cells gave rise to certain mechanisms that partially impeded antigen-specific immune responses, hindering the immune system’s ability to effectively eliminate tumors. Furthermore, investigations had revealed a noteworthy correlation between the quantity of tumor-associated macrophages (TAMs) and the progression as well as prognosis of prostate cancer. In mouse models of prostate cancer, a profuse infiltration of TAMs had been found to be linked with the advancement of tumor growth. In mice expressing HLA-DR2b, TAMs were found in excessive numbers and may have exhibited immunosuppressive effects. Conversely, in mice that mounted a robust immune response against PSA, TAMs were relatively scarce [17–19].

Previous research had indicated that increased levels of CD33 might have contributed to an elevated risk of prostate cancer. Tumor cells upregulated Siglec ligands carrying sialic acid, which could interact with Siglec expressed on CD33-bearing cells, thereby suppressing immune cell activation. Additionally, LGALS3BP, as a heavily glycosylated secreted molecule, was upregulated during tumor progression and bound to human Siglec-9 and other immune-modulatory Siglecs [20]. Therefore, the abnormal expression of CD33 and LGALS3BP might have collaboratively facilitated the development of prostate cancer and immune evasion. This suggested that tumor cells could have evaded immune system attacks by interacting with Siglecs expressed on CD33-bearing cells, thus contributing to an increased risk of prostate cancer. CD14, a glycoprotein, is expressed on the surface of monocytes and macrophages. It plays a crucial role in innate immunity and antigen presentation. Previous studies have indicated that changes in CD14 expression and function are associated with immune response dysregulation in the context of PCa. CD14+ monocytes obtained from PCa patients were found to exhibit immunosuppressive properties. These monocytes inhibited leukocyte proliferation and suppressed the expression of proinflammatory cytokines and HLA class II molecules. Additionally, increased levels of CD4+CD25high regulatory T (Treg) cells, known for their potent immunosuppressive effects, were observed in both the blood and tumor tissue of early-stage PCa patients. These Treg cells directed monocyte differentiation into an alternatively activated phenotype characterized by anti-inflammatory effects and immunosuppression.

This study aimed to thoroughly investigate the causal associations between 731 immune cell signatures and PCa by utilizing genetic instruments selected from the largest and most up-to-date GWAS datasets for these diseases, using a Two-sample MR design. Various sensitivity analyses were conducted to account for potential pleiotropic biases, and the MR results were validated for their robustness. Additionally, it is worth mentioning that all participants included in the GWAS were individuals of European ancestry, thereby minimizing the possibility of population stratification bias distorting the study findings. The consistent genetic predisposition of immune cell signatures with PCa across two different data sources and various MR models suggests that the results obtained were unlikely to be influenced by horizontal pleiotropy. However, there were several limitations to consider when interpreting our research findings. Firstly, a significant amount of Cochran’s Q statistic data suggested the presence of heterogeneity. To address this issue, we selected the IVW random-effects method as our main MR approach, which had been shown to be reliable (NO32). Another concern was overfitting, as both GWAS data sources had overlapping samples and features. However, we believed that our results were not misleading since we obtained the venous injection from a large-scale GWAS. Thirdly, despite conducting multiple sensitivity analyses, we were unable to fully assess horizontal pleiotropy. Fourthly, due to a lack of personal information, we could not perform further population stratification analysis. Fifthly, our study was based on a European database and may not be generalizable to other ethnic groups. Sixthly, some common risk factors for prostate cancer were not adjusted for due to the large number of analyses conducted. Lastly, we could not estimate the interactions between genetic and environmental factors through aggregated genetic statistics, highlighting the need for further validation studies to explore the associations of other risk factors with prostate cancer.

## CONCLUSIONS

This study utilized MR and GWAS summary statistics to investigate the causal relationship between 731 immune cell features and PCa. The main findings of this study demonstrated a significant association between three immune cell features, namely HLA DR on CD33+ HLA DR+ CD14dim, HLA DR on CD33+ HLA DR+ CD14−, and HLA DR on monocyte, and the risk of PCa. Although there is no significant causal relationship between PCa and higher proportion of immune cell features, these results are still of great significance for further understanding the underlying mechanisms of PCa. By revealing the association between immune cell features and PCa, we can further explore the role of the immune system in the development of PCa, aiding in the development of novel preventive and therapeutic strategies. However, despite contributing to our understanding of PCa, this study still raises several questions that need to be addressed. For instance, our study is solely based on genomic associations and statistical analyses, thus requiring additional experimental and clinical research to validate these results and explore the potential clinical applications of immune cell features in PCa screening, diagnosis, and treatment.

## Supplementary Material

Supplementary Figures

Supplementary Table 1

Supplementary Table 2

Supplementary Table 3

Supplementary Table 4

Supplementary Table 5

Supplementary Table 6

Supplementary Table 7

Supplementary Table 8
