# Supplementary material for: Causal role of immune cells in prostate cancer: a bidirectional Mendelian-randomization analyses
Source: Aging (Albany NY). 2024 Jun 17;16(12):10477–88. doi: 10.18632/aging.205942 (PMC11236311; doi:10.18632/aging.205942)
Supplement: Supplementary Figures [file aging-16-205942-s001.pdf]

SUPPLEMENTARY FIGURES

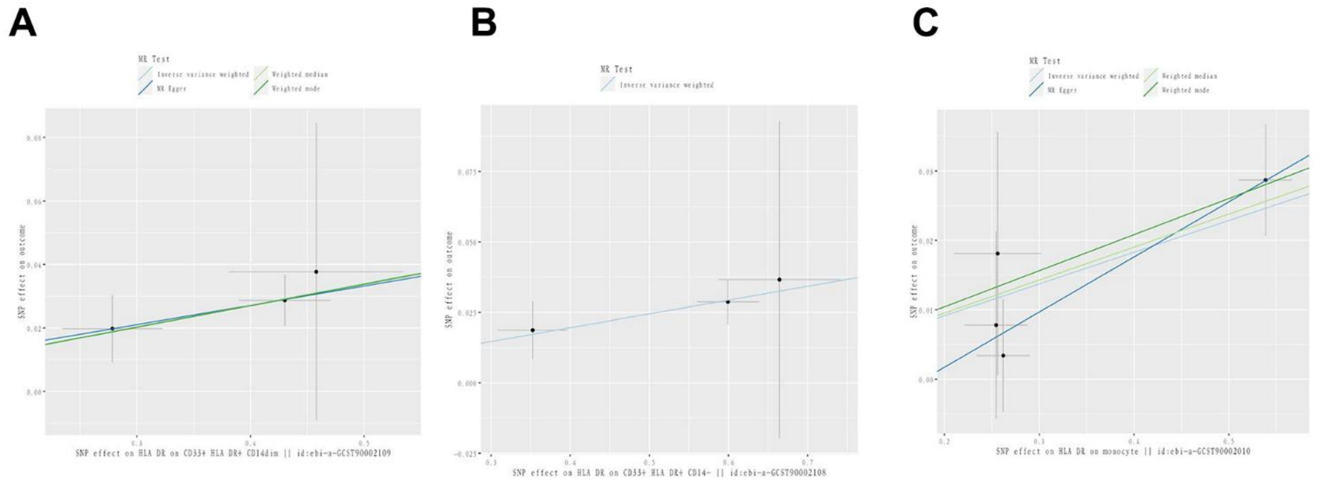

Supplementary Figure 1. The scatter plots for the Mendelian randomization immune cells-to-prostate cancer. (**A**) HLA DR on CD33+ HLA DR+ CD14dim, (**B**) HLA DR on CD33+ HLA DR+ CD14−, (**C**) HLA DR on monocyte.

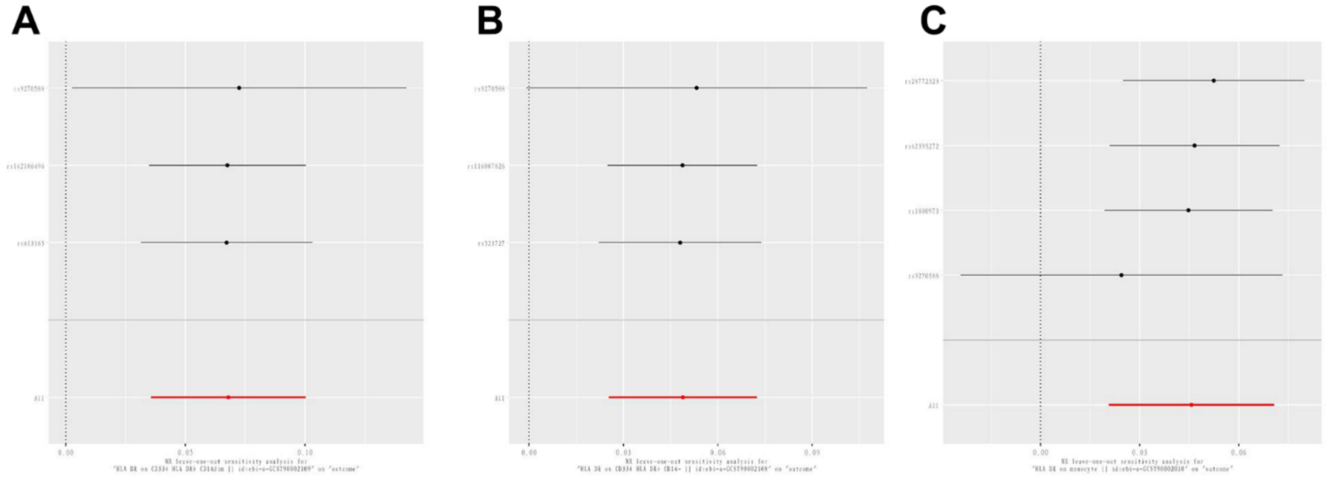

Supplementary Figure 2. The leave-one-out sensitivity analysis between prostate cancer and immune cells. (**A**) HLA DR on CD33+ HLA DR+ CD14dim, (**B**) HLA DR on CD33+ HLA DR+ CD14−, (**C**) HLA DR on monocyte.
